# Supplementary material for: Combining polygenic risk scores to understand genetic liability to physical-mental health multimorbidity in UK Biobank
Source: Hum Mol Genet. 2026 Apr 27;35(7):ddag012. doi: 10.1093/hmg/ddag012 (PMC13116345; doi:10.1093/hmg/ddag012)
Supplement: Supplementary_materials_ddag012 [file supplementary_materials_ddag012.zip › 2026-01-30_LINC_PGS_Supplemental.docx]

**Supplemental materials: TABLE OF CONTENTS**

[Supplemental table 1: Characteristics of participants in the complete UKB sample and the subset eligible for this study 2](#_Toc220919620)

[Supplemental table 2: Characteristics of GWAS used to create PRS_TRAIT_ 3](#_Toc220919621)

[Supplemental figure 1: Combinations of LINC conditions in 39,311 UKB (of 206,452) participants with ICM-MM 6](#_Toc220919622)

[Supplemental figure 2: Associations between PRS_TRAIT_ and internalising and cardiometabolic traits, and ICM-MM 7](#_Toc220919623)

[Supplemental figure 3: Elastic net retained PRS_TRAIT_ weights and performance metrics for ICM-MM in the testing sample 8](#_Toc220919624)

[Supplemental figure 4: Correlations between PRS_TRAIT_ and PRS_GPLC_ 9](#_Toc220919625)

[Supplemental figure 5: Odds ratios for all PRS and ICM-MM in n=44,600 UKB participants 10](#_Toc220919626)

[Supplemental figure 6: AUC for all PRS and ICM-MM in n=44,600 UKB participants 11](#_Toc220919627)

[Supplemental figure 7: Weights for PRS_GPLC_ and prediction metrics in the 50% testing subset 12](#_Toc220919628)

[Supplemental figure 8: Weights for PRS_TRAIT_ and prediction metrics in the GPLC subset 13](#_Toc220919629)

[PRS_TRAIT_ GWAS references 14](#_Toc220919630)

[Supplemental codelist information 15](#_Toc220919631)

##

## Supplemental table 1: Characteristics of participants in the complete UKB sample and the subset eligible for this study

|  | **UKB Total** | **Excluded** | **Included** |
| --- | --- | --- | --- |
|  | (N=502182) | (N=295730) | (N=206452) |
| **Gender** |  |  |  |
| Female | 273185 (54.4%) | 161232 (54.5%) | 111953 (54.2%) |
| Male | 228997 (45.6%) | 134498 (45.5%) | 94499 (45.8%) |
| **Year of Birth** |  |  |  |
| Median [Q1,Q3] | 1950 [1950,1960] | 1950 [1950,1960] | 1950 [1950,1960] |
| **Townsend deprivation score** |  |  |  |
| Median [Q1,Q3] | -2.14 [-3.64,0.550] | -2.12 [-3.63,0.620] | -2.16 [-3.65,0.460] |
| Missing | 624 (0.1%) | 311 (0.1%) | 313 (0.2%) |
| **Ethnicity** |  |  |  |
| Asian or Asian British | 9872 (2.0%) | 5683 (1.9%) | 4189 (2.0%) |
| Black or Black British | 8049 (1.6%) | 5868 (2.0%) | 2181 (1.1%) |
| Chinese | 1573 (0.3%) | 1023 (0.3%) | 550 (0.3%) |
| Do not know | 217 (0.0%) | 138 (0.0%) | 79 (0.0%) |
| Mixed | 2951 (0.6%) | 1894 (0.6%) | 1057 (0.5%) |
| Other ethnic group | 4553 (0.9%) | 3003 (1.0%) | 1550 (0.8%) |
| Prefer not to answer | 1660 (0.3%) | 1047 (0.4%) | 613 (0.3%) |
| White | 472409 (94.1%) | 276332 (93.4%) | 196077 (95.0%) |
| Missing | 898 (0.2%) | 742 (0.3%) | 156 (0.1%) |
| **Anxiety** |  |  |  |
| Prevalence | NA | NA | 32443 (15.7%) |
| Age onset (Median [Q1,Q3]) | NA | NA | 51.0 [42.0,60.0] |
| **Depression** |  |  |  |
| Prevalence | NA | NA | 49457 (24.0%) |
| Age onset (Median [Q1,Q3]) | NA | NA | 49.0 [40.0,58.0] |
| **Somatoform disorders** |  |  |  |
| Prevalence | NA | NA | 7995 (3.9%) |
| Age onset (Median [Q1,Q3]) | NA | NA | 52.0 [44.0,59.0] |
| **Hypertension** |  |  |  |
| Prevalence | NA | NA | 79633 (38.6%) |
| Age onset (Median [Q1,Q3]) | NA | NA | 59.0 [52.0,66.0] |
| **Obesity** |  |  |  |
| Prevalence | NA | NA | 29565 (14.3%) |
| Age onset (Median [Q1,Q3]) | NA | NA | 59.0 [51.0,67.0] |
| **Dyslipidemia** |  |  |  |
| Prevalence | NA | NA | 58560 (28.4%) |
| Age onset (Median [Q1,Q3]) | NA | NA | 61.0 [54.0,67.0] |
| **T2D** |  |  |  |
| Prevalence | NA | NA | 20240 (9.8%) |
| Age onset (Median [Q1,Q3]) | NA | NA | 62.0 [56.0,69.0] |
| **CKD** |  |  |  |
| Prevalence | NA | NA | 17126 (8.3%) |
| Age onset (Median [Q1,Q3]) | NA | NA | 66.0 [60.0,70.0] |

Condition specific prevalences using codes present in primary care (Read code) and Hospital Episode Statistics (HES – ICD codes). UKB Total and Excluded prevalences NA because primary care data is currently available for ~45% of the total sample (‘Included’)

## Supplemental table 2: Characteristics of GWAS used to create PRS_TRAIT_

| **Reference + data URL / doi** | **Study [contributing data sources highlighted in bold]** | **Trait** | **Sample size** | **Ancestries** | **N cases** | **Variance explained in out of sample prediction** |
| --- | --- | --- | --- | --- | --- | --- |
| [(Wray et al. 2018)](https://paperpile.com/c/NvT1LG/FGu1)  10.6084/m9.figshare.21655784 | Psychiatric Genomics Consortium (PGC) - Meta GWAS of seven cohorts. Summary statistics used highlighted in bold)  **PGC29, deCODE, GenScotland, GERA, iPSYCH**,  UK Biobank,  23andMe (discovery sample) | Depression | 143,265 | European | 45,591 | 1.9% (variance explained on liability scale, for full meta analysis) |
| [(Meier et al. 2019)](https://paperpile.com/c/NvT1LG/8x28) | Lundbeck Foundation Initiative for Integrative Psychiatric Research (iPSYCH) study | Anxiety | 31,880 | European | 12,655 | 28% (0.28, SE 0.027) |
| No citation found | - | Somatoform disorder | - | - | - | - |
| [(Mahajan et al. 2018)](https://paperpile.com/c/NvT1LG/A8zQ)  <https://diagram-consortium.org/downloads.html>  T2D GWAS meta-analysis - Summary of T2D associations, unadjusted for BMI and without UK Biobank subjects | DIAbetes Genetics Replication And Meta-analysis consortium (DIAGRAM): Meta analysis of 32 GWAS (provided sumstats excluding UKB) **BioME**  **deCODE**  **DGDG**  **DGI**  **EGCUT_ExomeCore**  **EGCUT_Human370CNV**  **EGCUT_OmniExpress**  **FHS**  **FUSION**  **GCKD**  **GENOA**  **GERA**  **GoDARTS**  **GOMAP-TEENAGE**  **HPFS**  **INTERACT_coreexome**  **INTERACT_GWAS**  **KORA**  **MESA**  **METSIM**  **MGI**  **NHS**  **NUGENE**  **PIVUS**  **PROSPER**  **RS1**  **RS2**  **RS3**  UK BioBank  **ULSAM**  **UPCH**  **WTCCC** | Type 2 diabetes | 455,313 | European | 55,005 | ~16.3% |
| [(Pattaro et al. 2016)](https://paperpile.com/c/NvT1LG/sMiz)  https://ckdgen.imbi.uni-freiburg.de/datasets/Pattaro_2016 | CKDGen: Meta-GWAS from 43 studies (excluding UKB) | Chronic kidney disease | 117,165 | European | 12,385 | 3.22% |
| [(Locke et al. 2015)](https://paperpile.com/c/NvT1LG/Fd4J)  https://giant-consortium.web.broadinstitute.org/images/1/15/SNP_gwas_mc_merge_nogc.tbl.uniq.gz | GIANT consortium: Meta GWAS of 114 studies (excluding UKB) | BMI (proxy for Obesity) | 322,206 | European | NA (quantitative trait) | 6.6% for SNPs with P<5x10^-3^, 21.6% for all HapMap3 SNPs |
| [(Willer et al. 2013)](https://paperpile.com/c/NvT1LG/bASa)  https://csg.sph.umich.edu/willer/public/lipids2013/ | Global Lipids Genetics Consortium Meta analysis of 45 studies (excluding UKB) | LDL-cholesterol | 188,577 | European, East Asian, South Asian and African ancestry | NA (quantitative trait) | 2.4% |
| [(Keaton et al. 2024)](https://paperpile.com/c/NvT1LG/SiH5)  <https://www.ncbi.nlm.nih.gov/projects/gap/cgi-bin/study.cgi?study_id=phs000585.v2.p1> | Meta analysis of 4 cohorts (Sumstats used in bold)  MVP  BioVU  UKB  **ICBP (Large meta-analysis of 70 studies)** | (Study reports Systolic and Diastolic blood pressure (SBP/DBP), and pulse pressure (PP), used here to proxy hypertension | 299,024 | European | NA (quantitative trait) | SBP 6.8% DBP 6.83% PP 4.29% |

##

## Supplemental figure 1: Combinations of LINC conditions in 39,311 UKB (of 206,452) participants with ICM-MM


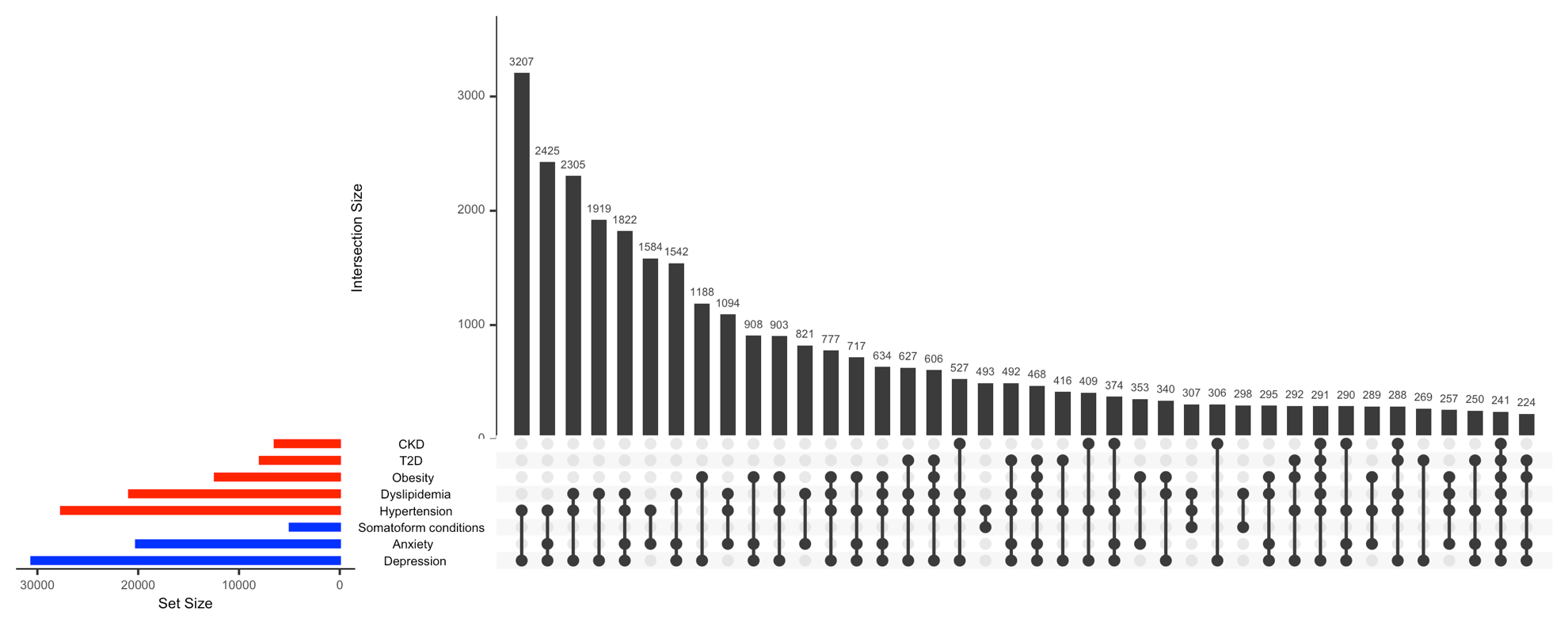


## Supplemental figure 2: Associations between PRS_TRAIT_ and internalising and cardiometabolic traits, and ICM-MM


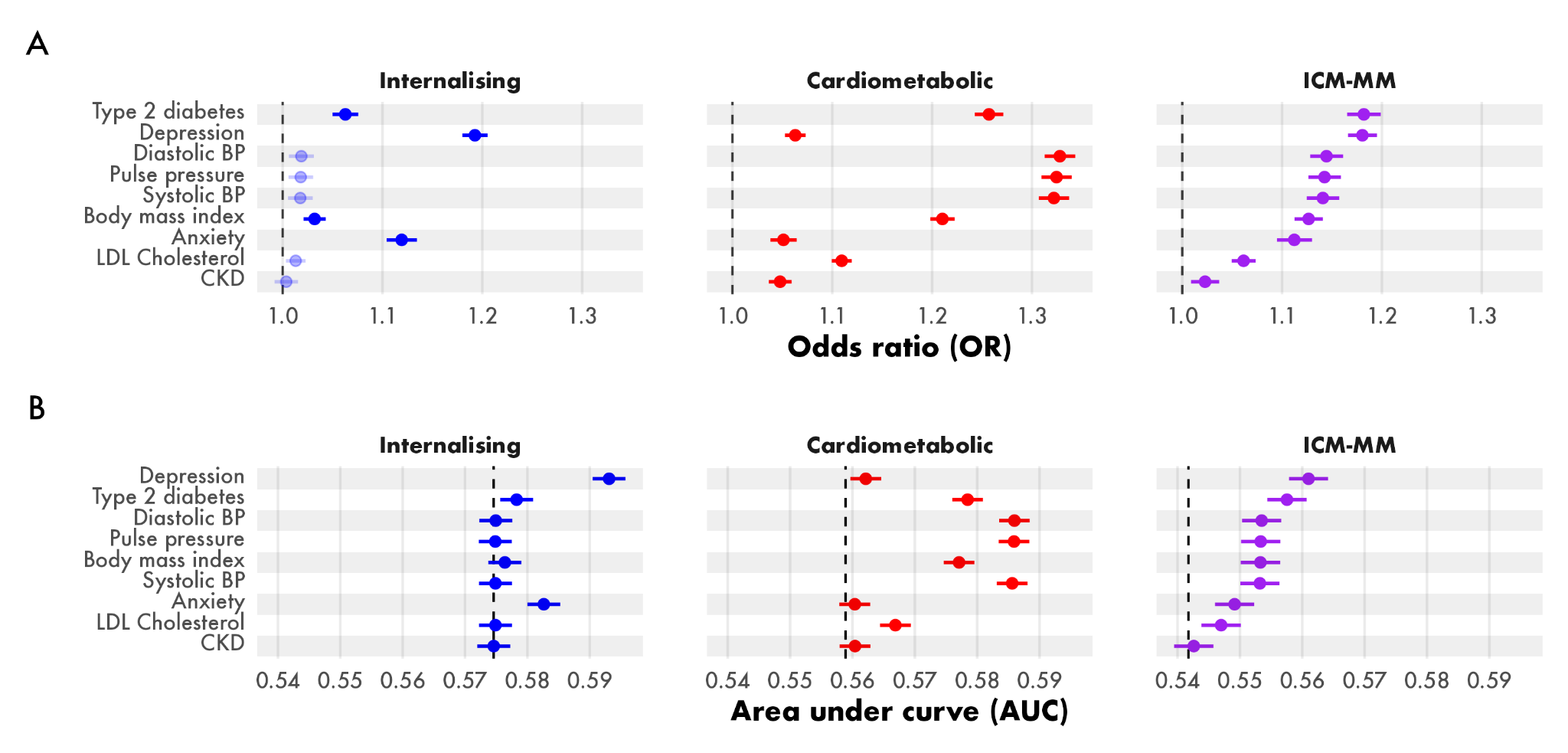


A: Odds ratios (OR) and 95% confidence intervals for effect size. Dashed line reference (OR=1). Highlighted results passed Bonferroni correction for multiple testing

B: Area under curve (AUC) and 95% confidence intervals: Dashed reference line for ‘null’ model (i.e. adjusted for self-reported gender and genetic PC covariates only)

## Supplemental figure 3: Elastic net retained PRS_TRAIT_ weights and performance metrics for ICM-MM in the testing sample


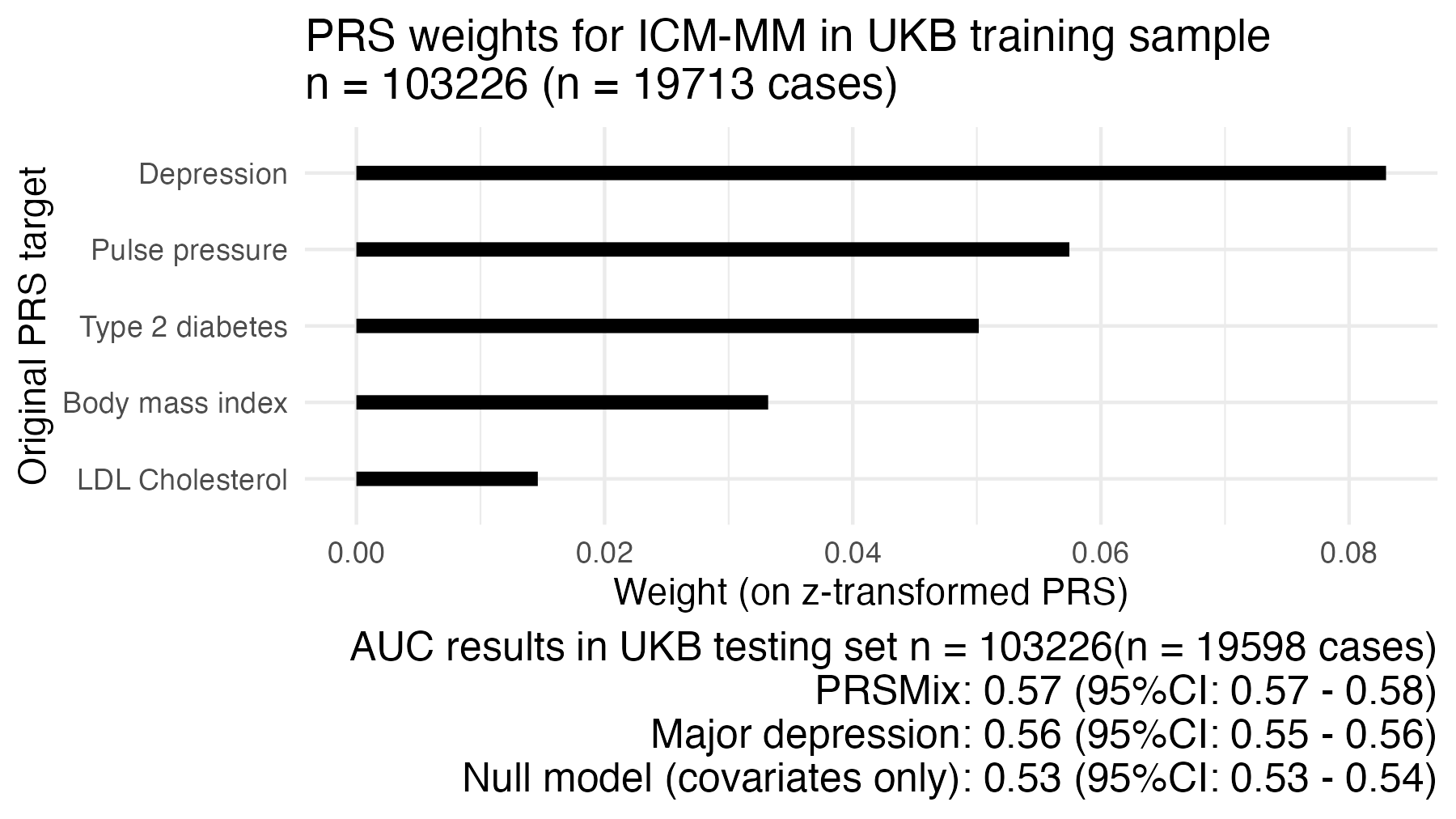


## Supplemental figure 4: Correlations between PRS_TRAIT_ and PRS_GPLC_


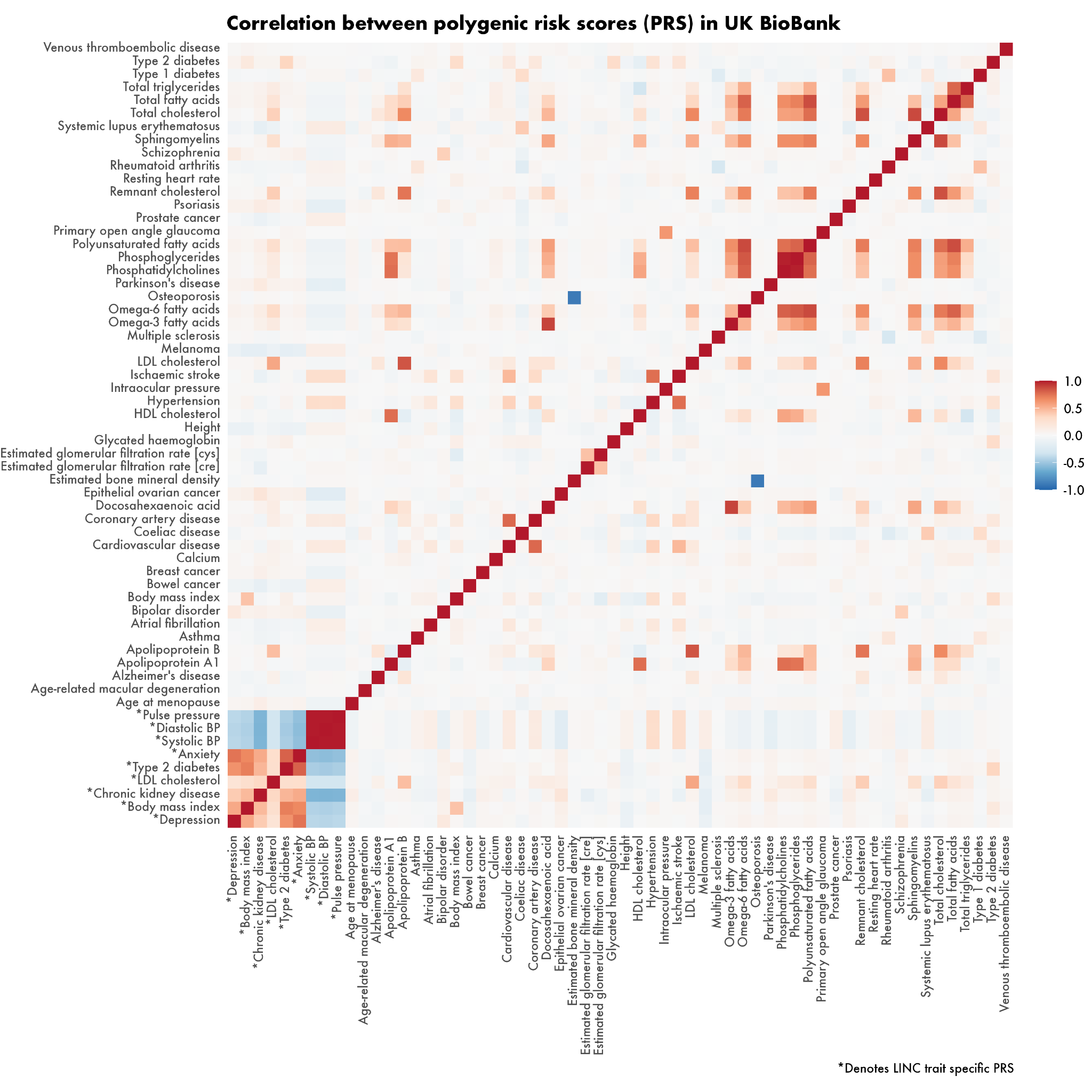


##

## Supplemental figure 5: Odds ratios for all PRS and ICM-MM in n=44,600 UKB participants


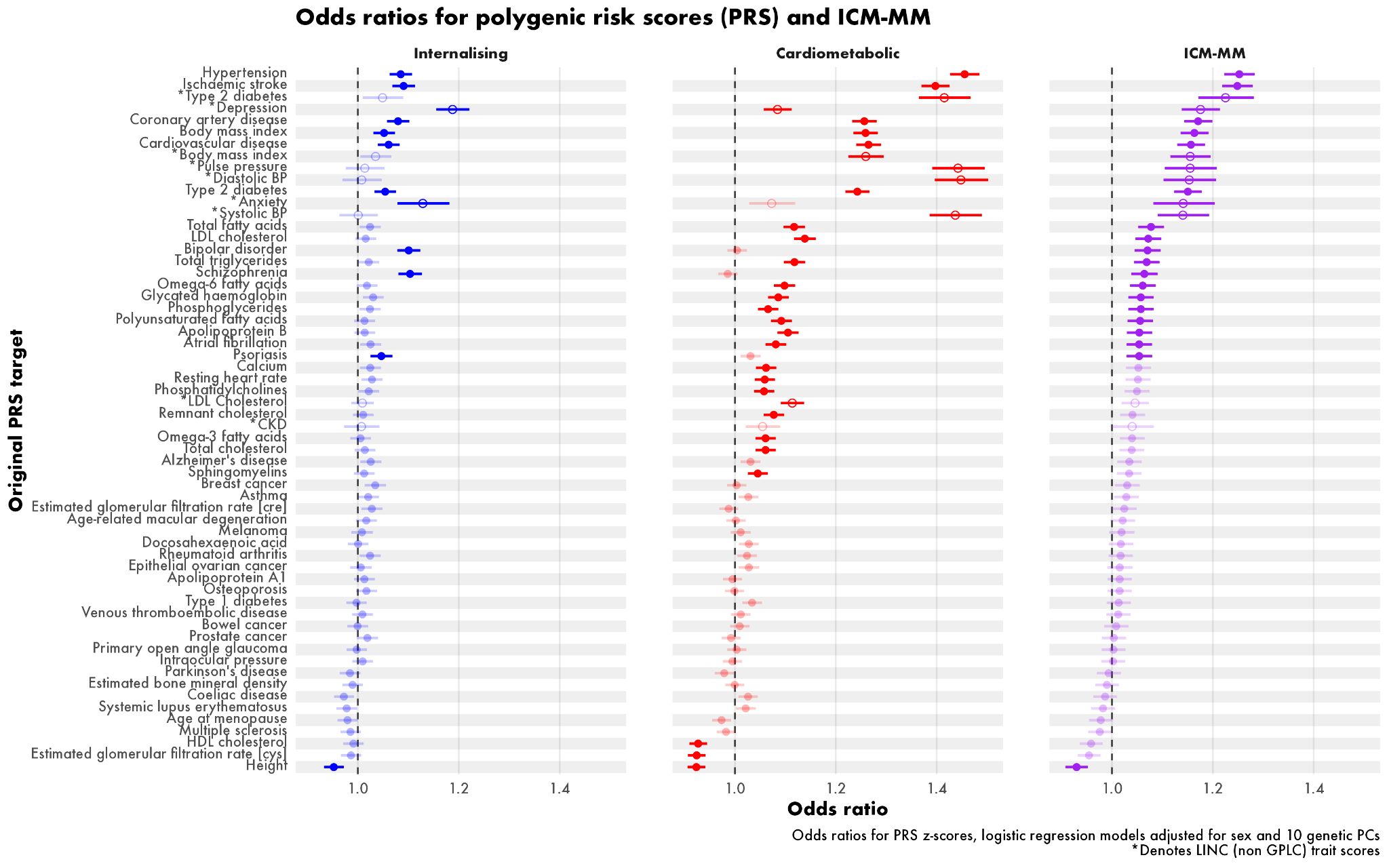


Odds ratios and associated 95% confidence intervals – highlighted estimates passed Bonferroni correction for multiple testing. Open circles denote LINC (non GPLC) trait scores. Dashed reference line for OR=1. This figure also includes PRS_TRAIT_ scores for comparison: some participants with PRS_GPLC_ did not meet the inclusion criteria for generating PRS_TRAIT_ hence the number here is lower than for the PRS_GPLC_ analysis n=45,493).

## Supplemental figure 6: AUC for all PRS and ICM-MM in n=44,600 UKB participants


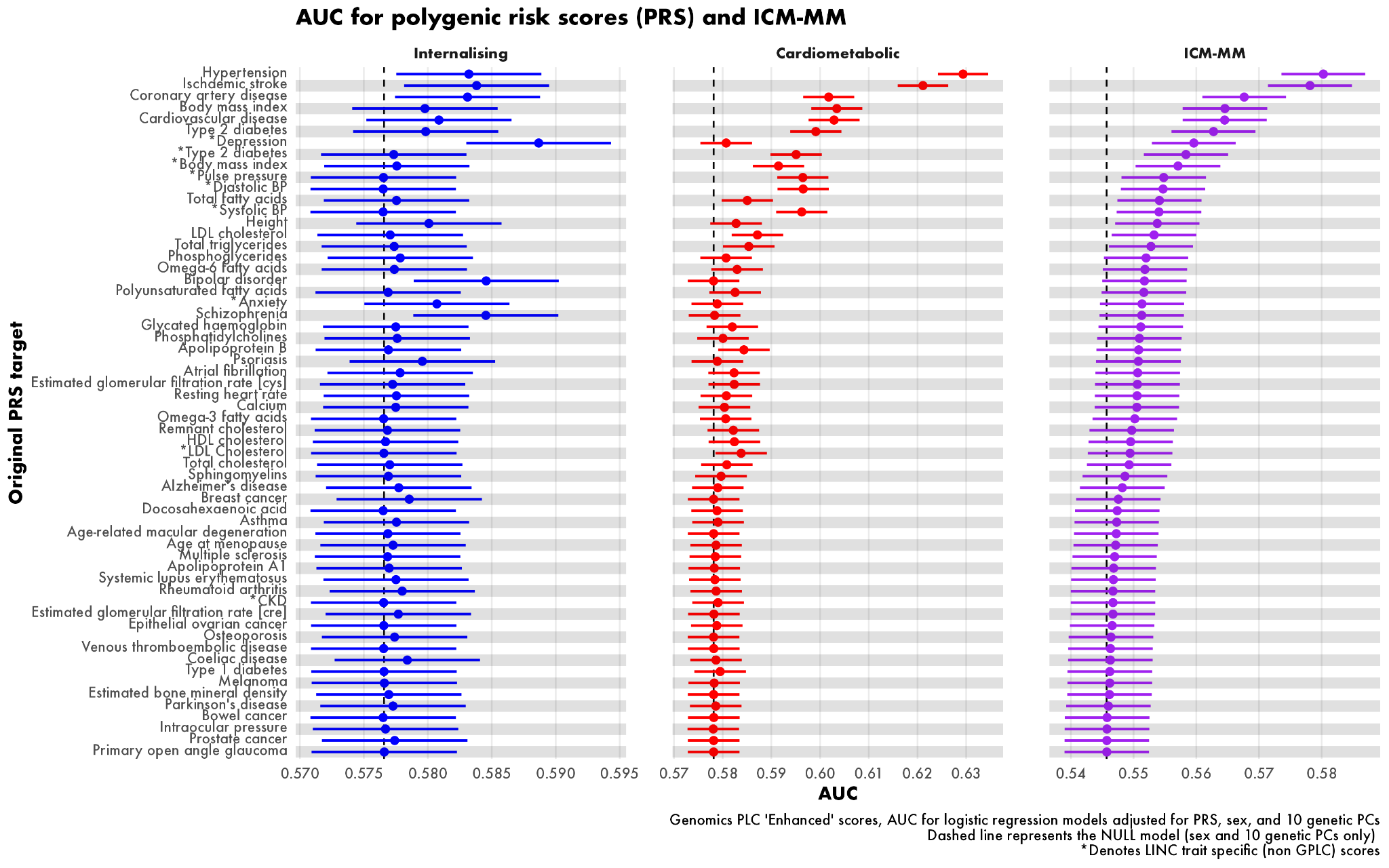


Area under the curve (AUC) and associated 95% confidence intervals. Dashed reference line for null model (adjusted for self-reported gender and genetic PC covariates only). Figure also includes PRS_TRAIT_ scores for comparison: some participants with PRS_GPLC_ did not meet the inclusion criteria for generating PRS_TRAIT_ hence the number here is lower than for the PRS_GPLC_ analysis n=45,493

## Supplemental figure 7: Weights for PRS_GPLC_ and prediction metrics in the 50% testing subset


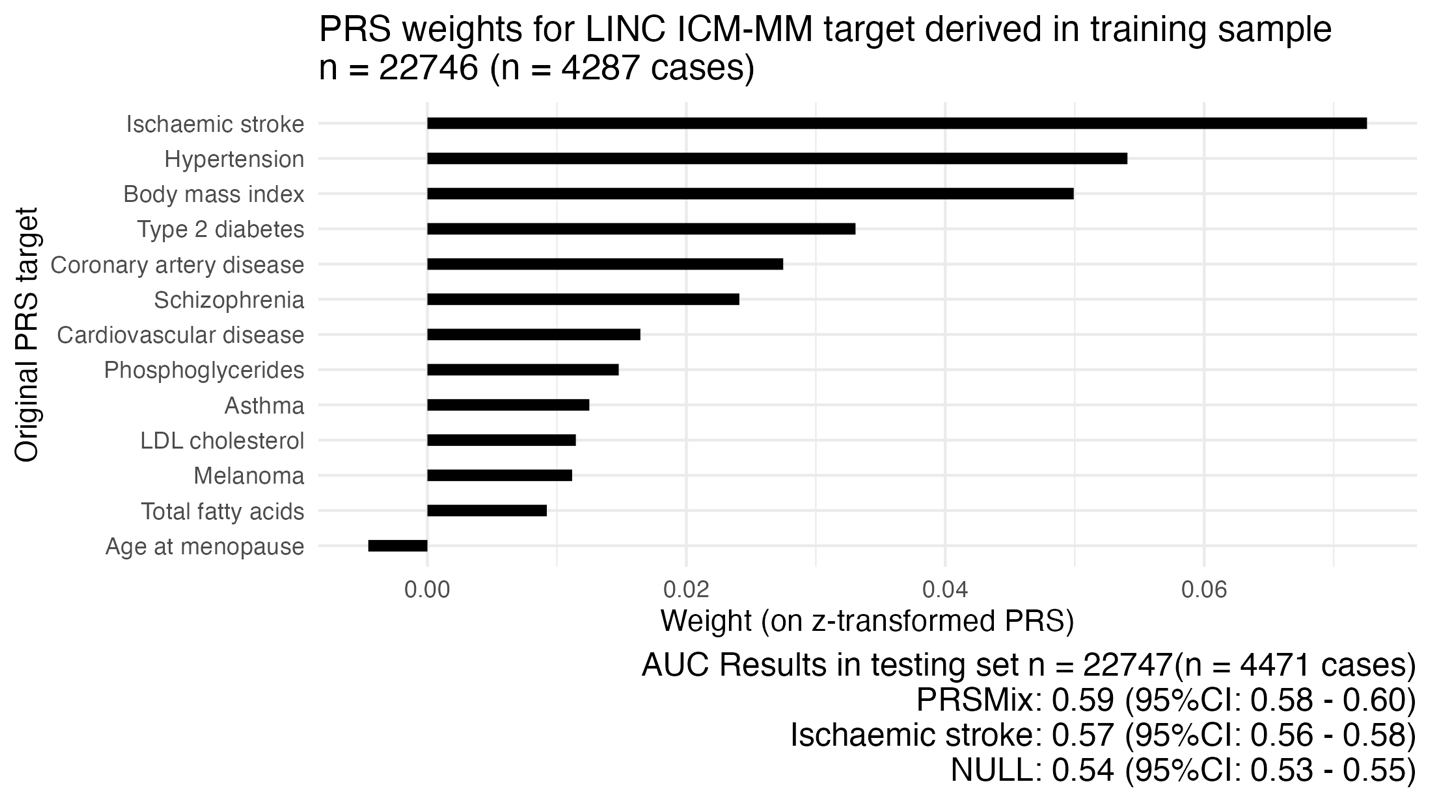


## Supplemental figure 8: Weights for PRS_TRAIT_ and prediction metrics in the GPLC subset


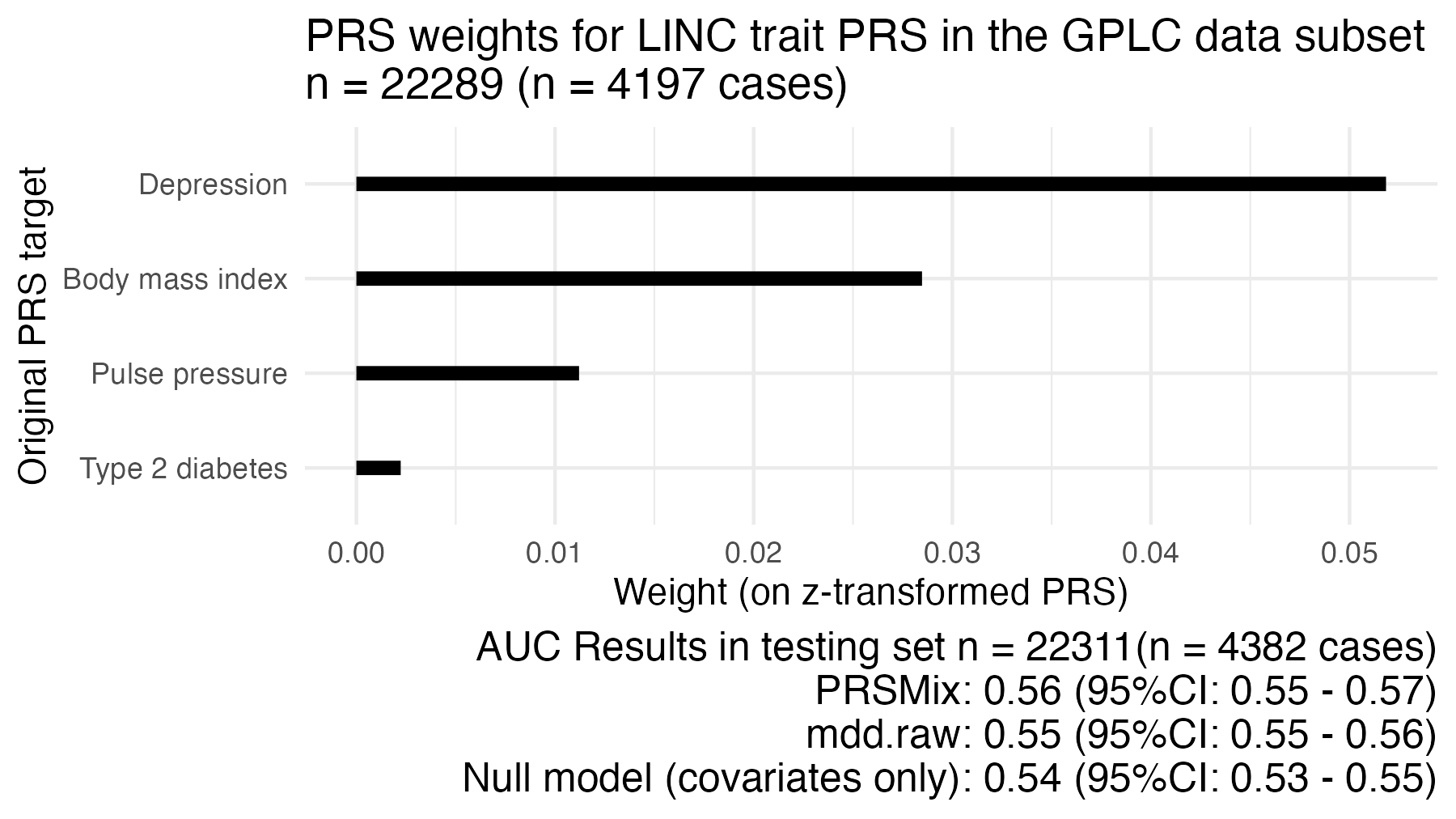


## PRS_TRAIT_ GWAS references

[Keaton, Jacob M., Zoha Kamali, Tian Xie, Ahmad Vaez, Ariel Williams, Slavina B. Goleva, Alireza Ani, et al. 2024. “Genome-Wide Analysis in over 1 Million Individuals of European Ancestry Yields Improved Polygenic Risk Scores for Blood Pressure Traits.” *Nature Genetics* 56 (5): 778–91.](http://paperpile.com/b/NvT1LG/SiH5)

[Locke, Adam E., Bratati Kahali, Sonja I. Berndt, Anne E. Justice, Tune H. Pers, Felix R. Day, Corey Powell, et al. 2015. “Genetic Studies of Body Mass Index Yield New Insights for Obesity Biology.” *Nature* 518 (7538): 197–206.](http://paperpile.com/b/NvT1LG/Fd4J)

[Mahajan, Anubha, Daniel Taliun, Matthias Thurner, Neil R. Robertson, Jason M. Torres, N. William Rayner, Anthony J. Payne, et al. 2018. “Fine-Mapping Type 2 Diabetes Loci to Single-Variant Resolution Using High-Density Imputation and Islet-Specific Epigenome Maps.” *Nature Genetics* 50 (11): 1505–13.](http://paperpile.com/b/NvT1LG/A8zQ)

[Meier, Sandra M., Kalevi Trontti, Kirstin L. Purves, Thomas Damm Als, Jakob Grove, Mikaela Laine, Marianne Giørtz Pedersen, et al. 2019. “Genetic Variants Associated with Anxiety and Stress-Related Disorders: A Genome-Wide Association Study and Mouse-Model Study: A Genome-Wide Association Study and Mouse-Model Study.” *JAMA Psychiatry (Chicago, Ill.)* 76 (9): 924–32.](http://paperpile.com/b/NvT1LG/8x28)

[Pattaro, Cristian, Alexander Teumer, Mathias Gorski, Audrey Y. Chu, Man Li, Vladan Mijatovic, Maija Garnaas, et al. 2016. “Genetic Associations at 53 Loci Highlight Cell Types and Biological Pathways Relevant for Kidney Function.” *Nature Communications* 7 (1): 10023.](http://paperpile.com/b/NvT1LG/sMiz)

[Willer, Cristen J., Ellen M. Schmidt, Sebanti Sengupta, Gina M. Peloso, Stefan Gustafsson, Stavroula Kanoni, Andrea Ganna, et al. 2013. “Discovery and Refinement of Loci Associated with Lipid Levels.” *Nature Genetics* 45 (11): 1274–83.](http://paperpile.com/b/NvT1LG/bASa)

[Wray, Naomi R., Stephan Ripke, Manuel Mattheisen, Maciej Trzaskowski, Enda M. Byrne, Abdel Abdellaoui, Mark J. Adams, et al. 2018. “Genome-Wide Association Analyses Identify 44 Risk Variants and Refine the Genetic Architecture of Major Depression.” *Nature Genetics* 50 (5): 668–81.](http://paperpile.com/b/NvT1LG/FGu1)

## Supplemental codelist information

**ICM-MM phenotype definitions in UKB primary and Hospital Episode Statistics (HES) data.**

UK Biobank primary care data use a mixture of Read2 and CTV3 clinical codes. To support phenotype definition using existing codelist resources, all CTV3 primary care codes were mapped to Read2 codes using UKB resource 592 ‘all_lkps_maps_v4.xlxs’ worksheet ‘read_v2_read_ctv3’

- Read2 codelists for ICM-MM conditions are available as **supplemental data file primary_icmm_codes.csv**
- Read2 codes for dyslipidemia were based on hypercholesterolaemia codes in Baksh r, et al., Multiple morbidity across the lifespan in people with Down syndrome or intellectual disabilities: a population-based cohort study using electronic health records. The Lancet Public Health, Volume 8, Issue 6, e453 - e462**:** <https://doi.org/10.1016/S2468-2667(23)00057-9> (for original lists: see their supplemental pp238 -240)

UK Biobank HES data use a mixture of ICD10 and ICD9 codes. To support phenotype definition using existing codelist resources, all ICD9 codes were mapped to ICD10 codes using UKB resource 592: ‘all_lkps_maps_v4.xlxs’ worksheet ‘icd9_icd10’.

- ICD10 codelists for ICM-MM conditions are available as supplemental data file **“secondary_icmm_codes.csv”**
- ICD10 codes for dyslipidemia were identified manually by clinicians working in the LINC collaborative: "E780", "E781", "E782", "E783", "E784", "E785","E8881"
